# Pulse oximetry screening for critical congenital heart disease in Tanzanian newborns: Diagnostic accuracy, sensitivity, and specificity in a low-resource healthcare setting

**DOI:** 10.1371/journal.pgph.0004904

**Published:** 2025-07-17

**Authors:** Naizihijwa Gadi Majani, Pilly Chillo, Mkiwa Akida, Judith Lamosai, Deogratias Nkya, Stella Mongella, Zawadi Kalezi, Godwin Sharau, Vivienne Mlawi, Peter Kisenge, Mohamed Janabi, Diederick Grobbee, Martijn Slieker

**Affiliations:** 1 Department of Pediatric Cardiology, Jakaya Kikwete Cardiac Institute, Dar es Salaam, Tanzania; 2 Julius Global Health, Julius Center for Health Sciences and Primary Care, Utrecht University, Utrecht, The Netherlands; 3 Department of Internal Medicine, Muhimbili University of Health and Allied Sciences (MUHAS), Dar es Salaam, Tanzania; 4 Department of Pediatrics, Mwananyamala Regional Hospital, Dar es Salaam, Tanzania; 5 Department of Pediatrics, Muhimbili National Hospital, Dar es Salaam, Tanzania; 6 Department of Pediatrics and Child Health, Muhimbili University of Health and Allied Sciences (MUHAS), Dar es Salaam, Tanzania; 7 Directorate of Surgery, Jakaya Kikwete Cardiac Institute, Dar es Salaam, Tanzania; 8 Department of Adult Cardiology, Jakaya Kikwete Cardiac Institute, Dar es Salaam, Tanzania; 9 Department of Pediatric Cardiology, Wilhelmina Children’s Hospital, Utrecht, The Netherlands; University of the Witwatersrand Johannesburg Faculty of Health Sciences, SOUTH AFRICA

## Abstract

Early detection of Critical Congenital Heart Disease (CCHD) is crucial for reducing infant mortality. Pulse oximetry (POX) is widely utilised for screening CCHD in high-resource settings; however, its diagnostic accuracy in low-resource environments, such as sub-Saharan Africa, remains under-researched. This study aimed to assess the diagnostic accuracy of POX in screening Tanzanian newborns for CCHD. This prospective cohort study was conducted in two hospitals in Dar es Salaam, Tanzania. We used pre- and post-ductal saturation (SpO2) readings prior to discharge. A positive screen was defined as SpO2 < 90%; two pre- and post-ductal SpO2 readings <95%; and/or a pre- or post-ductal difference that exceeded 3%. All newborns with positive screening tests underwent echocardiography, while those with negative tests were followed for six months. The primary outcome was POX diagnostic accuracy. The study adhered to STARD guidelines for reporting diagnostic accuracy studies. Between October 2020 and June 2023, 10,630 newborns were screened. The majority (5,721; 54.0%) were male, resulting in a male-to-female ratio of 1.2. The median birth weight was 3.0 (IQR: 2.6–4.4) kg. A total of 51 (0.5%) newborns tested positive on POX, of which 18 (35.3%) had congenital heart disease (CHD), and 15 (83.3%) were classified as critical, leading to a CCHD prevalence of 1.41 per 1,000 live births (95% CI: 0.70–2.13), which increased to a cumulative prevalence of 3.27 per 1,000 live births (95% CI: 2.29–4.67) at six months. With a follow-up rate of 86.7% (9,170/10,574), POX demonstrated a sensitivity of 50.0% (95% CI: 32.1–67.9), a specificity of 99.5% (95% CI: 99.4–99.7), a false-positive rate of 0.4%, and an overall accuracy of 99.5% (95% CI: 99.2–99.5). Screenings conducted between 48 and 72 hours exhibited the highest diagnostic performance, AUC 0.79 (95% CI: 0.64–0.93), with a significant odds ratio (OR) of 5.31 (95% CI: 2.45–11.49, p = 0.00001). Newborns with a birth weight <2.5 kg were less likely to have CCHD detected by POX, OR 0.403 (95% CI: 0.19–0.87, p = 0.021). POX demonstrated lower sensitivity but higher specificity and diagnostic accuracy after 48 hours. The timing of screening and birth weight affected its accuracy, indicating a need for protocol adjustment.

## Introduction

Congenital heart disease (CHD) is the most common congenital anomaly globally, affecting approximately 8–12 per 1,000 live births [1]. Of these cases, around 25% (2.5 to 3.0 per 1,000 live births) are classified as Critical Congenital Heart Disease (CCHD), which requires urgent intervention or stabilization during the neonatal period [2].

In low-resource settings, particularly in sub-Saharan Africa (SSA), undiagnosed or late-diagnosed CCHD significantly contributes to neonatal mortality [3–7]. The absence of comprehensive screening programmes, such as prenatal echocardiography, along with limited access to advanced diagnostics like postnatal echocardiography and gaps in newborn care, delays timely diagnosis and treatment [3,5]. Moreover, the true prevalence of CCHD in SSA remains poorly documented, making it difficult to fully assess the extent of the problem [1,5]. The lack of robust follow-up mechanisms exacerbates this issue, as missed or delayed diagnoses frequently go unaddressed, leading to preventable deaths [3,8–10]. Bridging these gaps is crucial for enhancing the region’s neonatal and childhood congenital heart disease (CHD) outcomes.

In high-income countries, pulse oximetry (POX) screening has become a highly effective tool for detecting CCHD, with a specificity of 99.8% and a sensitivity of 78.4% [11–13]. Numerous studies have demonstrated that its widespread adoption has significantly reduced the number of undiagnosed CCHD cases, leading to earlier interventions and improved neonatal outcomes [14–16]. As a result, POX screening is now standard practice in newborn care, playing a critical role in identifying life-threatening heart conditions before they cause severe complications or death [16,17].

Given the success of POX in high-resource environments, it is imperative to evaluate its diagnostic performance and long-term impact in low-resource settings. Factors such as differences in birth practices, neonatal care, malnutrition, infections, and limitations of healthcare infrastructure may affect the sensitivity and specificity of POX screening in these settings [18,19]. Therefore, tailoring screening protocols to address the unique challenges faced in low-resource environments is critical [20,21]. In addition, understanding the prevalence of CCHD and long-term survival outcomes in these regions is vital for shaping public health strategies aimed at reducing neonatal and infant mortality [22,23].

We performed a prospective cohort study among newborns in Dar es Salaam, Tanzania, with the objectives of evaluating the effectiveness of pulse oximetry as a screening tool for CCHD in newborns.

## Methods

### Study design and study setting

This newborn pulse oximetry cohort study was conducted in two government-owned hospitals, Muhimbili National Hospital (MNH) and Mwananyamala Regional Hospital (MRH), located in Dar es Salaam, 24 meters above sea level by the Indian Ocean, the largest city in Tanzania with a population of over 5 million. The full protocol for this study was previously published [24]. Briefly, the study was conducted from October 4, 2020, to June 30, 2023, at MNH and from June 1, 2022, to June 30, 2023, at MRH, during which 10,630 newborns were screened. MNH is the tertiary national referral hospital and typically handles around 7,000 deliveries annually, featuring a NICU, a kangaroo mother care unit, and a 100-bed neonatal ward. MRH is a secondary care facility located 15 kilometres from MNH, managing approximately 6,000 annual deliveries. It also has a NICU, a kangaroo mother care unit, and a 30-bed neonatal ward. Follow-up for newborns diagnosed with CCHD was conducted at Jakaya Kikwete Cardiac Institute (JKCI), a specialised cardiac centre within the MNH grounds with a capacity of 157 beds. JKCI, currently the sole cardiac center for children, is well-equipped and capable of stabilising newborns with CCHD using Prostin and the Rashkind procedure before surgery. Since 2015, over 2,500 children have undergone cardiac surgeries at JKCI.

### Routine newborn care at MNH and MRH

Most mothers (98%) in the Dar es Salaam region deliver in a hospital [25]. Postnatal newborn examinations and femoral pulse palpation are not routine practices at either hospital. In a well-baby nursery, newborns are nursed with their mothers for observation, regularly weighed, and receive the first dose of immunization before discharge under the supervision of nursing staff. If a nurse suspects that a baby is not well based on visual assessment, or if the mother reports concerns, a medical doctor is consulted.

### Study site preparation

Before screening began, the study sites were evaluated for readiness, including the availability of staff and equipment. Antenatal ward nurses received a two-hour training session, followed by a competency test that required a score of 95% or higher. Eligible nurses were listed, and those who did not pass the test received feedback and were encouraged to retake it. The study team visited the sites daily to ensure adherence to the protocol, provide support, and collect questionnaires.

### Inclusion and exclusion criteria

All newborns who met the inclusion criteria were systematically enrolled and screened. These included all full-term or near-term newborns (gestational age > 35 weeks) delivered at the two hospitals and admitted to regular newborn nurseries. Newborns delivered before 35 weeks, or those admitted to the NICU due to severe illness or malformations, were excluded, as these infants undergo extensive investigations and are less likely to be overlooked for CCHD evaluation. The study team regularly visited the NICU to check for any CCHD diagnoses before discharge.

### Enrollment and pulse oximetry screening (index test)

Eligible newborns were enrolled for pulse oximetry (POX) screening within 12 hours of delivery or before discharge, with informed consent from the mother.

The screening process adhered to the U.S. Newborn Screening Program protocol [12], utilizing the Bistos BT-720 device (Bistos, Korea) to measure oxygen saturation (SpO₂) in the right hand and foot. A positive result was defined as SpO₂ < 90%, or pre- and post-ductal SpO₂ < 95% in two separate readings taken an hour apart, or a pre-/post-ductal difference greater than 3%. This POX test is a painless procedure with no anticipated adverse effects. After screening, a sticker was placed on the newborn’s postnatal card to facilitate follow-up if CCHD was diagnosed. POX-positive newborns without a CCHD diagnosis were referred to a paediatrician, while POX-negative newborns were contacted six months later to monitor for any subsequent diagnoses.

### Echocardiography evaluation (Reference standard)

Newborns with positive POX results underwent detailed echocardiography (ECHO) assessments at JKCI using a high-resolution Vivid E95 machine (GE Healthcare, USA), equipped with P-8 probes for newborns and M-5 probes for older children. The ECHO examinations were conducted by pediatric cardiologists and reviewed by a senior pediatric cardiologist. This procedure is painless and has no expected adverse effects. For this study, the definition of CCHD was based on standards from the American Academy of Pediatrics, American Heart Association, and CDC [12]. The study focused on 12 specific CCHD cases, categorized into three major groups with similar characteristics: ductal-dependent obstructive lesions, conotruncal lesions, and single-ventricle lesions. Additional details on the classifications are provided in S1 Appendix. Newborns diagnosed with CCHD were observed for 12 months, with follow-ups scheduled at 6 weeks, 6 months, and 12 months. Pediatric cardiologists at JKCI also monitored new CCHD cases presented at Muhimbili National Hospital (MNH) or Muhimbili Reference Hospital (MRH) during the study period.

### Sample size

The initial sample size was calculated to be 30,000 newborns, assuming a CCHD prevalence of 3 per 1,000 live births, with a sensitivity of 75% and specificity of 99.5%. This design aimed to provide 80% power to detect a sensitivity of at least 52% and 90% power to detect a specificity greater than 99.3%, with a one-sided α value of 2.5% [24]. However, due to resource and logistical constraints, the study enrolled a total of 10,630 newborns. A post-hoc power analysis indicated that with 10,630 screened newborns, the study achieved 88.9% power to detect a sensitivity of at least 50%. For specificity, the study was adequately powered, contributing to the specificity estimate of 99%.

### Data handling and statistical analysis

Data cleaning and quality control were conducted through consistent checks, followed by coding. Lastly, the data was entered into Redcap version 16 and analyzed using SPSS version 29.0.1.0. The reporting form recorded the infant’s age at screening, sex, birth weight, and delivery mode. Continuous variables were analyzed using means and standard deviations (SD) for normally distributed data and medians with interquartile ranges (IQR) for skewed data. Categorical variables were summarized as frequencies and percentages. The primary outcome was the diagnostic accuracy of POX in detecting CCHD. The study adhered to the Standards for Reporting of Diagnostic Accuracy Studies (STARD) guidelines, reporting diagnostic accuracy metrics, including sensitivity, specificity, predictive values, 95% confidence intervals, ROC curves, and calibration plots [26]. Patients missed during follow-up were regarded as missing not at random (MNR), and complete case analyses were employed.

## Results

### Demographic characteristics of screened newborns

**Fig 1:**
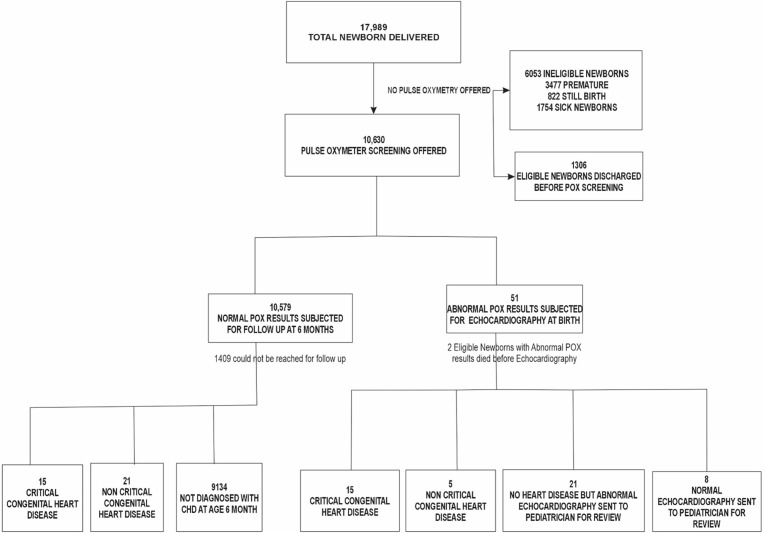
Process through pulse oximetry screening for critical congenital heart disease.

Between October 2020 and June 2023, there were 17,989 deliveries between MNH and MRH. At MNH, there were 13,949 deliveries (77.5%) and at MRH, 4,040 deliveries (22.5%). About 3,477 deliveries (19.3%) were preterm (< 35 weeks), 822 deliveries (4.6%) resulted in stillbirths, and 1,754 deliveries (9.7%) were for sick newborns admitted to NICU before screening, leaving 11,936 eligible for screening. A total of 10,630 newborns (89.0%) were screened for CCHD with POX, with missed screenings attributed to early discharge and insufficient staffing (Fig 1).

Males contributed to 5,721 newborns (54.0%) with a male-to-female ratio of 1.2, while caesarean section deliveries contributed to 5,721 (58.5%). The median birth weight was 3.0 kg (IQR: 2.6–4.4 kg). Mothers had a median age of 30 years (IQR: 25–34), with 95.1% (10,004) residing in Dar es Salaam (Table 1).

**Table 1:** Demographic characteristics of babies (N = 10,630).

Characteristic	Categories	N (%)
**Gestational Age:** weeks	Median (IQR)	38 (36–39)
**Birth Weight:** kg	Median (IQR)	3 (2.60–3.40)
**Birth Heigh:** cm	Median (IQR)	47 (45–49)
**Sex**	Male	5721 (54.0)
**Mode of Delivery**	Spontaneous vertex Delivery	4400 (41.4)
	Caesarean Section	6201 (58.5)
	Breach Delivery	24 (0.3)
	Undocumented	3 (0.0)
**Apgar Score at 5 Minutes**	1–4	34 (0.7)
	5–8	3624 (34.5)
	9–10	6864 (64.8)
**Obvious Congenital Malformation**	Yes	6 (0.6)
**System affected by Congenital Malformation**	GIT	12 (24.5)
	CNS	11 (22.4)
	GUS	4 (8.2)
	Skeletal Malformation	5 (10.2)
	Spinal Bifida	7 (14.3)
	Multi organ involvement	10 (20.4)
**Obvious Genetic Syndrome**	Yes	14 (0.1)

IQR = Interquartile Range; GIT = Gastrointestinal tract; CNS = Central nervous system. GUS = Genital Urinary System.

### POX screening results

The median age at first screening was 24 hours (IQR; 18–37), and 34 newborns (0.3%) required a second screening at a median age of 37 hours (IQR; 14–71). Of the 51 who tested positive on POX results, a total of 49 newborns (0.5%) were referred for echocardiography. Two newborns with positive POX results died before undergoing echocardiography and were excluded from further analysis. In most cases, POX tests and echocardiography were conducted within 24 hours, except for weekend deliveries, where the time frame was extended to 48–72 hours (Table 2).

**Table 2:** Summary of pulse oximetry screening results (N = 10,630).

Characteristic	Categories	N (%)
**Age in hours at Screening**	Hours: Median (IQR)	24 (18–37)
**First Screening SPO2**	SPO2 Right hand: Median (IQR)	97 (90–97)
	SPO2 Foot: Median (IQR)	96 (91–97)
**First Pulse Oximeter Result**	Positive Screen	48 (0.4)
	Negative Screen	10547(99.2)
	Undetermined results	34 (0.3)
**Age at Second Screening**	hours: Median (IQR)	37 (14–71)
**Second Screening SPO2**	SPO2 Right hand: Median (IQR)	95 (90–97)
	SPO2 Foot: Median (IQR)	95 (91–97)
**Second Pulse Oximeter Result**	Positive Screen	3 (8.8)
	Negative Screen	31(91.2)

IQR= Interquartile Range; SPO2= Peripheral Capillary Oxygen Saturation

### Echocardiography evaluation results

Echocardiography confirmed CCHD in 15 of the 49 babies, resulting in a birth prevalence of 1.41 (95% CI 0.70–2.13) per 1000 live births. Of the 34 false positives (0.45%), 8 (23.5%) were normal, while 26 (76.5%) had other conditions requiring urgent medical intervention [specifically, non-critical congenital heart defects (5; 19.2%), respiratory disorders (10; 38.5%), and infections (11; 42.3%)]. Among the 10,579 with negative POX screen results, 9170 (86.7%) were available for follow-up. Follow-up revealed an additional 15 CCHD cases, increasing the six-month cumulative prevalence for CCHD to 3.27 per 1,000 live births (95% CI: 2.29–4.67) (Fig 1).

### Accuracy of the pulse oximetry test and the associated factors

Table 3 shows the diagnostic performance of POX for CCHD. Of the 30 confirmed CCHD cases, 15 were detected by POX (true positive), while 15 were missed (false negative). There were 34 false positives and 9,155 true negatives, leading to a sensitivity of 50.0% (95% CI; 32.1–67.9) and a specificity of 99.5% (95% CI; 99.4–99.7). The diagnostic odds ratio (DOR) was 221.5 (100.4-488.6), with an overall accuracy of 99.5% (95% CI, 99.2– 99.5). The performance across three subgroups of CCHD varied, with the obstructive ductal dependency subgroup showing the highest sensitivity of 71.4% (95% CI; 33.9-92.4) compared to 45.0% (95% CI; 66.8-88.6) for cono-truncal lesions and 33.3% (95% CI; 20.01-86.7) for single ventricle lesions. Significant factors linked to screening performance included the time of screening and birth weight. Newborns screened between 48–72 hours were significantly more likely to be accurately identified with CCHD (OR=5.31; 95% CI: 2.45–11.49, p = 0.00001), than those screened under 48 hours; and those with a birth weight below 2.5 kg were less likely to be detected by POX (OR=0.403; 95% CI: 0.186–0.873, p = 0.021). Although newborns delivered by SVD demonstrated a tendency to be accurately identified by POX (OR=1.613; 95% CI: 0.73–3.54), it did not significantly impact the accuracy (p = 0.234; S2 Appendix and S3 Appendix).

**Table 3:** Accuracy of pulse oximetry in detection of Critical Congenital Heart Disease (N = 9170).

Characteristic	Critical Cases: (N = 9170:TP = 15; FP = 34; TN = 9106; FN = 15) Proportion (%)	95% CI	For all CHD: (N = 9170:TP = 20; FP = 29; TN = 9065; FN = 56) Proportion (%)	95% CI
**Sensitivity**	50.0	32.1–67.9	26.3	16.4–36.2
**Specificity**	99.6	99.5–99.8	99.7	99.6–99.8
**Positive Predictive Value**	30.6	17.7–43.5	40.8	27.1–54.6
**Negative Predictive Value**	99.8	99.8–99.9	99.4	99.2–99.6
**Percentage Accuracy**	99.5	98.3–99.6	99.1	98.9–99.3
**False Positive rate**	0.4	0.3–0.5	0.3	0.2–0.4
**Sensitivity Cono-truncal**	71.4	33.9–91.9		
**Sensitivity Obstructive Lesions**	45.0	66.8–86.6		
**Sensitivity Single ventricle Physiology lesions**	33.3	20.0–86.7		

TP= True Positive; FP= False Positive; TN= True Negative; FN=False Negative

## Discussion

This study evaluated the diagnostic accuracy of pulse oximetry screening (POX) for detecting critical congenital heart disease (CCHD) in a low-resource setting in Tanzania with a cohort of over 10,000 newborns. Pulse oximetry demonstrated a high specificity of **99.5%**, consistent with findings from studies in high-resource settings [11–14]. However, the sensitivity was **50%**, significantly lower than the global average of 76.3% [27]. This reduced sensitivity aligns with findings from other studies conducted in similar low-resource settings in southeast Asia, southern Africa, and northern Africa, where sensitivity typically ranges from 20% to 50% [28–31]. The discrepancy in sensitivity likely reflects the unique contextual challenges of resource-constrained environments, including limited access to reliable equipment, shortages of trained personnel, and barriers to follow-up care [18–22]. In contrast, high-resource settings benefit from more advanced neonatal care, better equipment, and standardized screening protocols [15]. Despite the lower-than-expected sensitivity, the findings of this study provide a critical foundation for optimizing screening protocols and integrating pulse oximetry into routine neonatal care in low-resource settings.

Moreover, our study emphasizes the critical influence of timing on the diagnostic performance of POX screening. We found that newborns screened 48–72 hours after birth were 5.31 times more likely to be correctly diagnosed with CCHD compared to those screened within 24–48 hours, with an Area Under the Curve (AUC) of 0.79. These findings are consistent with prior research showing improved sensitivity when POX screening is conducted later, as delayed screening allows for the resolution of transitional circulatory changes that may mask hypoxemia [11,13,16]. Early screening, within the first 24–48 hours, may result in false positives and false negatives due to physiological changes such as ductal closure, which can obscure the detection of hypoxemia [32].

While delaying screening to 48–72 hours could enhance detection rates, we acknowledge that this must be weighed against the risk of missing early diagnoses, particularly in severe CCHD cases that may deteriorate within the first 24–48 hours [12,33]. Indeed, two POX-positive newborns in our study passed away before echocardiography could be conducted, with CCHD being highly suspected, highlighting the potential risks associated with delayed follow-up. These instances underscore the necessity of implementing robust systems for immediate follow-up and timely referral for urgent cases whenever there is a delay in screening. Considering these factors, a dual screening strategy might provide a balanced solution, as suggested in the literature [31,32]. This approach involves an initial screening within the first 24 hours of life to identify early critical cases, followed by a second screening at 48–72 hours to enhance overall detection rates. Nevertheless, executing such a strategy in resource-limited settings presents logistical challenges, including staffing limitations and delays in follow-up testing [18]. Additional research is necessary to assess the practicality, advantages, and disadvantages of dual screening strategies in these contexts, emphasizing the balance between early detection and logistical challenges.

Furthermore, our study revealed that newborns delivered vaginally were more likely to have CCHD detected (OR = 1.61), while low birth weight (<2.5 kg) notably decreased detection likelihood (OR = 0.40, p = 0.021). This indicates that low birth weight significantly predicts a reduced effectiveness of POX. Infants with low birth weight are susceptible to respiratory distress and various neonatal complications, which may interfere with POX readings and result in increased false negatives. Additionally, hemodynamic variations in these infants may affect POX’s ability to detect hypoxemia linked to CCHD. Vaginal deliveries showed higher sensitivity than CS deliveries, possibly due to differences in neonatal transition physiology. Further research is needed to understand the decreased sensitivity in low-birth-weight infants and develop tailored screening protocols. Possible improvements include enhanced monitoring, adjusted POX thresholds, or supplementary echocardiography for high-risk infants. Customizing screening by birth weight and delivery mode is key to better CCHD detection, especially in resource-limited settings.

Notably, the relatively low sensitivity observed in our study highlights the need for broader improvements in newborn screening in Tanzania and similar settings. For instance, only 49% of newborns in Tanzania are examined within two days of birth, which means many newborn illnesses, including CHD, go undetected [24]. Although this study did not directly examine the practice of newborn physical examinations at the study hospitals, it is common for newborns to be evaluated only if parents or nurses raise concerns following an uncomplicated delivery. Routine newborn examination, as recommended by the WHO, is crucial in the early identification of CCHD and other conditions [34].

In addition to identifying CCHD, POX flagged other life-threatening conditions requiring urgent intervention, including respiratory and infectious diseases. Combining POX with a thorough physical examination has been shown to increase CCHD detection sensitivity to as much as 92%, particularly for non-hypoxic forms of CHD [27,35]. These non-hypoxic cases, which constitute the majority, are often treatable even in low-resource settings. Therefore, ensuring that every newborn receives a comprehensive physical examination while training healthcare workers to recognize early signs of CHD is essential for early detection and could significantly reduce neonatal mortality [6,8]. Moreover, our study indicates that most late-diagnosed CHD cases were detected at an average age of two months, underscoring the need to extend screening efforts to include routine childhood visits, such as during immunization [20–23].

In summary, while POX identified only 50% of CCHD cases at birth, its high specificity resulted in a low rate of false positives (0.4%), thereby reducing the need for unnecessary procedures. Notably, 74.5% of cases flagged by POX in our study required urgent medical attention for non-cardiac issues, highlighting POX’s broader potential as a screening tool for serious unrelated conditions. Ultimately, our results highlight both the strengths and weaknesses of POX in detecting CCHD in resource-limited environments, while also indicating how factors such as timing, delivery method, and birth weight influence screening outcomes.

### Strengths and limitations

This study has certain limitations yet presents important strengths, particularly in low-resource environments. One limitation is that some patient outcomes were not recorded due to unsuccessful follow-up calls, leading to Missing at Random (MAR) data. We mitigated this issue by conducting a Complete Case Analysis and carrying out a sensitivity analysis to validate the robustness of our findings. Notably, we achieved a commendable follow-up rate of 86.7%. A significant strength of our research is the inclusion of Mwananyamala, a regional hospital that reflects the conditions evident in 28 other regional hospitals across Tanzania. This aspect enhances the generalizability of our results, suggesting applicability to similar hospitals nationwide. However, the findings may have limited relevance for lower-level health centers with constrained diagnostic infrastructures, particularly those lacking echocardiography capabilities, or in high-altitude regions where protocols may require adaptation. Inherent in a smaller number of positive cases limited the precision of the sensitivity estimate, as reflected by the wide confidence interval (32.1% to 67.9%). However, the specificity estimate was highly precise (98.8% to 99.2%), underscoring the reliability of the test in identifying CCHD cases. Nonetheless, our work provides a robust foundation for future innovations aimed at improving CCHD detection and neonatal care in low-resource settings.

### Study contributions

This study is one of the largest assessments of POX for CCHD in sub-Saharan Africa and one of the few that explores its diagnostic performance under real-world conditions in a low-resource setting. It provides novel insights into:

1. The optimal timing of screening in settings with high early discharge rates.
2. The impact of delivery method and birth weight on screening outcomes.
3. The potential of POX to detect non-cardiac conditions in addition to CCHD.

### Recommendations and future research

This study’s findings offer several essential recommendations for enhancing CCHD screening in low-resource environments. Future research should validate these findings in larger, multicenter cohorts to improve statistical robustness; focus on refining screening protocols, particularly the practicality of dual screening methods—an initial assessment within the first 24 hours, followed by a second evaluation at 48–72 hours—to effectively identify both early and late-onset cases. Investigating the role of artificial intelligence (AI) in augmenting POX accuracy and overcoming resource limitations is beneficial.

Although this study did not specifically evaluate cost-effectiveness, the low cost of POX devices and their minimal training requirements suggest that POX screening may provide a cost-effective solution for routine neonatal care in low-resource settings. Nevertheless, further research is needed to explore the cost-effectiveness of dual screening protocols in low-resource environments, to thoroughly assess cost-effectiveness and confirm the long-term viability of large-scale POX deployment in similar contexts.

## Conclusion

Pulse oximetry screening for CCHD demonstrated high specificity but moderate sensitivity in this Tanzanian cohort. Screening timing, delivery method, and birth weight influenced diagnostic performance. Despite its limitations, this study underscores the importance of adapting POS protocols to local contexts and highlights its broader utility in improving neonatal outcomes in low-resource settings. Continued research and refinement of screening protocols are essential to optimize implementation and impact.

### Ethics approval

The Muhimbili University of Health and Allied Sciences approved this study (application No MUHAS-REC-2-2020-082) with the Ethics Committee’s consent. Further permission was obtained from the National Institute of Medical Research (NIMR) under application No NIMR/HQ/R.8a/Vol. III/96. Additional authorisation was sought from Muhimbili National Hospital and the Jakaya Kikwete Cardiac Institute. The study adhered to the ethical guidelines of the 1975 Declaration of Helsinki for research involving human subjects.

**What’s known**

1. Pulse oximetry is a validated screening tool for Critical Congenital Heart Disease (CCHD) in newborns. It demonstrates high specificity (99.8%) but moderate sensitivity (78.5%) (40).
2. Data on the diagnostic accuracy, feasibility, and impact of pulse oximetry screening in low-resource settings are limited (3, 20, 21).

**What’s new**

1. This study provides evidence from a low-resource setting in Tanzania, East Africa, indicating that pulse oximetry has low sensitivity (50%) and high specificity (99.5%) for CCHD detection. It emphasizes the need to integrate other screening modalities to enhance sensitivity.
2. It emphasizes the importance of timing to improve diagnostic accuracy and suggests protocol adjustments, particularly for low-birth-weight infants, to increase the detection rate.

**Impact**

This study reinforces the vital importance of pulse oximetry in saving newborn lives by detecting critical congenital heart disease (CCHD) early, especially in resource-limited settings. It highlights the need for protocol improvements to enhance sensitivity and advocates for the integration of complementary screening methods. By promoting advancements in neonatal care, this research has the potential to transform early detection strategies and significantly reduce infant mortality in underserved regions.

## Supporting information

S1 Appendix Definitions of Critical Congenital Heart Disease (CCHD) and classification of primary and secondary target lesions for pulse oximetry screening. Includes standard international definitions adapted from AHA and AAP guidelines.
(DOCX)

S2 Appendix Factors affecting the diagnostic accuracy of pulse oximetry screening in newborns. Displays odds ratios, confidence intervals, and statistical significance for key demographic and clinical variables.
(DOCX)

S3 Appendix Sensitivity analysis of diagnostic accuracy across subgroups. Includes timing of screening, mode of delivery, and newborn characteristics with corresponding AUC and confidence intervals.
(DOCX)

S4 Appendix STARD 2015 Checklist: Adherence to the Standards for Reporting of Diagnostic Accuracy Studies. (PDF)

S5 Appendix Inclusivity in Global Research Questionnaire: Tool used to evaluate equity considerations in the design and implementation of the study. (PDF)

S6 Appendix Anonymized raw dataset used for analysis in this study: Newborn screening for CCHD in Tanzania. (CSV)
