# Supplementary material for: Pulse oximetry screening for critical congenital heart disease in Tanzanian newborns: Diagnostic accuracy, sensitivity, and specificity in a low-resource healthcare setting
Source: PLOS Glob Public Health. 2025 Jul 17;5(7):e0004904. doi: 10.1371/journal.pgph.0004904 (PMC12270164; doi:10.1371/journal.pgph.0004904)
Supplement: S1 Appendix — Includes standard international definitions adapted from AHA and AAP guidelines. (DOCX) [file pgph.0004904.s001.docx]

**S1 Appendix**: Definitions of Terms for CCHD

Critical Congenital Heart Disease: All lesions requiring surgery or catheter-based intervention within the first year of life to prevent death or severe disability. Typically results in hypoxemia and can be detected with pulse oximetry (POX). These include definitions as offered by AAP and AHA, which are the 7 Primary and 5 secondary target conditions.

**Primary Targets for POX newborn screening**

1. Hypoplastic Left Heart Syndrome (HLHS)
2. Pulmonary Atresia (with intact interventricular septum)
3. Tetralogy of Fallot (TOF)
4. Total Anomolous Pulmonary venous return (TAPVR)
5. Transposition of Great Arteries (TGA)
6. Tricuspid Atresia
7. Truncus Arteriosus

**Secondary Targets for POX newborn screening**

1. Coarctation of Aorta (CoA)
2. Double Outlet Right Ventricle (DORV)
3. Severe Aortic Stenosis
4. Ebstain Anomaly
5. Interrupted Aortic Arch

**Group 1:** Cono truncal lesions

1. Tetralogy of Fallot
2. Truncus Arteriosus
3. Double Outlet Right Ventricle

**Group 2:** Obstructive Ductal Dependent Lesions

1. Hypoplastic Left Heart Syndrome
2. Coactation of Aorta
3. Pulmonary Atresia
4. Critical Aortic Stenosis
5. Critical Pulmonary Stenosis

**Group 3:** Single ventricle Physiology

1. Tricuspid Atresia
2. Single ventricle
3. Double Inlet Left Ventricle
