# Supplementary material for: Pulse oximetry screening for critical congenital heart disease in Tanzanian newborns: Diagnostic accuracy, sensitivity, and specificity in a low-resource healthcare setting
Source: PLOS Glob Public Health. 2025 Jul 17;5(7):e0004904. doi: 10.1371/journal.pgph.0004904 (PMC12270164; doi:10.1371/journal.pgph.0004904)
Supplement: S2 Appendix — Displays odds ratios, confidence intervals, and statistical significance for key demographic and clinical variables. (DOCX) [file pgph.0004904.s002.docx]

**S2 Appendix**: Factor affecting Diagnostic accuracy

| **Factor** |  | | **95% Wald Confidence Interval** | | **P-value** | **Key intepretation** |
| --- | --- | --- | --- | --- | --- | --- |
|  | **Odds Ratio (OR** ) | **Std. error** | **Lower** | **Upper** | **Significance** |  |
| **Age at Screening**  **48-72 hours** | 5.531 | 0.286 | 3.155 | 6.969 | <0.001* | **Optimal screening window** |
| **Mode of Delivery (SVD)** | 1.613 | 0.402 | 0.734 | 3.546 | 0.234 | Non significant difference |
| **Low birth weight(<2.4kg)** | 0.403 | 0.393 | 0.186 | 0.871 | 0.021* | Significantly reduced detection |
| **Seks (Female)** | 1.071 | 0.374 | 0.515 | 2.229 | 0.854 | Minimal non significant difference |

**Significant P-value*
