# Supplementary material for: Pulse oximetry screening for critical congenital heart disease in Tanzanian newborns: Diagnostic accuracy, sensitivity, and specificity in a low-resource healthcare setting
Source: PLOS Glob Public Health. 2025 Jul 17;5(7):e0004904. doi: 10.1371/journal.pgph.0004904 (PMC12270164; doi:10.1371/journal.pgph.0004904)
Supplement: S3 Appendix — Includes timing of screening, mode of delivery, and newborn characteristics with corresponding AUC and confidence intervals. (DOCX) [file pgph.0004904.s003.docx]

**S3 Appendix:** Sensitivity Analysis on Diagnostic Accuracy

| ***Subgroup*** | ***Sensitivity*** | ***95% CI*** | ***AUC*** | ***95% CI*** | ***Sample size (n1/n2)*** | ***P-value*** |
| --- | --- | --- | --- | --- | --- | --- |
| ***Overall*** | *50.00* | *32.11-67.89* | *0.748* | *0.630-0.860* | *30/9170* | *0.00..* |
| ***Timing of Screening*** |  |  |  |  |  |  |
| *24-48 hours* | *38.58* | *26.30-38.50* | *0.690* | *0.51-0.790* | *13/3006* | *0.039* |
| *49-72 hours* | *58.80* | *38.50-68.80* | *0.788* | *0.642-0.934* | *17/1373* | *0.004* |
| ***Mode of Delivery*** |  |  |  |  |  |  |
| *Vaginal (SVD)* | *66.70* | *59.23-71.45* | *0.831* | *0.643-1.020* | *9/4351* | *0.001* |
| *Caesarean (C-section)* | *42.90* | *36.00-54.60* | *0.713* | *0.572-0.758* | *21/6239* | *0.003* |
| ***Body weight*** |  |  |  |  |  |  |
| *Normal and Low weight* | *50.00* | *32.11-67.89* | *0.749* | *0.549-0.949* | *30/9130* | *0.015* |
| ***By sex*** |  |  |  |  |  |  |
| *Male* | *52.90* | *29.56-62.80* | *0.763* | *0.612-0.914* | *17/5721* | *0.001* |
| *Female* | *46.20* | *33.30-49.90* | *0.730* | *0.552-0.907* | *13/4877* | *0.011* |
